# Total Intravenous Versus Inhalational Anesthesia in High-Grade Glioma Surgery: A Systematic Review and Meta-Analysis

**DOI:** 10.3390/medicina61081463

**Published:** 2025-08-14

**Authors:** Plamen Penchev, Boris Tablov, Mariano Gallo Ruelas, Daniela Milanova-Ilieva, Lyubomir Gaydarski, Nikolay Yordanov, Eduardo Alonso, Danna Espinoza, Petar-Preslav Petrov, Ivelina Lukanova, Pavel Stanchev, Julian Dichev, Ivana Korentova, Nikolai Ramadanov

**Affiliations:** 1 Faculty of Medicine, Medical University of Plovdiv, 4002 Plovdiv, Bulgaria; sonaonetrick@abv.bg (P.P.);; 2 Department of Anesthesiology and Intensive Care, Multiprofile Hospital for Active Treatment “Life Hospital”, 8008 Burgas, Bulgaria; 3 Instituto de Investigación Nutricional (IIN), Lima 15024, Peru; 4 Department of Pediatrics, Medical University of Plovdiv, 4002 Plovdiv, Bulgaria; 5 Department of Anatomy, Histology and Embryology, Medical University of Sofia, 1431 Sofia, Bulgaria; 6 Department of Neurointensive Care, Multiprofile Hospital for Active Treatment in Neurology and Psychiatry “St. Naum”, 1113 Sofia, Bulgaria; 7 Hospital de Especialidades Teodoro Maldonado Carbo, Universidad Católica de Santiago de Guayaquil, Guayaquil 090615, Ecuador; 8 Department of Anatomy, Histology and Embryology, Medical University of Plovdiv, 4002 Plovdiv, Bulgaria; 9 Faculty of Medicine, Medical University, 1000 Sofia, Bulgaria; 10 Clinic of Endocrinology and Metabolic Diseases, St. George University Hospital, Medical University of Plovdiv, 4002 Plovdiv, Bulgaria; 11 Faculty of Medicine, Trakia University, 6000 Stara Zagora, Bulgaria; 12 Center of Orthopaedics and Traumatology, Brandenburg Medical School, University Hospital Brandenburg, 14770 Brandenburg an der Havel, Germany; 13 Faculty of Health Science Brandenburg, Brandenburg Medical School Theodor Fontane, 16816 Brandenburg an der Havel, Germany

**Keywords:** high-grade gliomas, glioblastoma, total intravenous anesthesia (TIVA), inhalational anesthesia (INHA)

## Abstract

*Background and Objectives*: High-grade gliomas (HGGs) are aggressive primary brain tumors with a poor prognosis despite multimodal treatment. The anesthetic technique used during surgery may influence tumor progression and survival, but its role in HGGs remains unclear. This meta-analysis evaluated the effect of total intravenous anesthesia (TIVA) versus inhalational anesthesia (INHA) on overall survival (OS) and progression-free survival (PFS) in HGG patients. *Materials and Methods*: A systematic search was conducted in PubMed, Scopus, and Cochrane databases for studies assessing the impact of TIVA versus INHA on OS and PFS in HGG patients. Statistical analysis was performed using R version 4.3.1. Heterogeneity across studies was quantified using the Cochrane Q test alongside the I^2^ statistic. A random-effects model was employed to derive the pooled hazard ratios (HRs). *Results*: A total of five studies involving 827 participants (mean age 58 years, mean females 38%) were included, of whom 406 (49%) received TIVA. No statistically significant differences were observed in OS (HR 0.77; 95% CI [0.58–1.02]; *p* = 0.07; I^2^ = 67%) or PFS (HR 0.88; 95% CI [0.70–1.10]; *p* = 0.27; I^2^ = 51%) between the groups. A subgroup analysis revealed that TIVA was associated with improved OS in patients with grade IV tumors (HR 0.70; 95% CI [0.51–0.96]; *p* = 0.03), while no significant effect was observed in the mixed grade III–IV subgroup. However, the test for subgroup differences was not statistically significant (*p* = 0.0669), and this finding should be interpreted with caution. No significant differences were observed in median OS or PFS, or in single-arm meta-analyses. *Conclusions*: This meta-analysis found no statistically significant differences in overall or progression-free survival between TIVA and INHA in patients undergoing surgery for HGGs. Although a subgroup analysis suggested a possible survival advantage of TIVA in grade IV tumors, the lack of a statistically significant subgroup difference test limits the strength of this finding. Further investigation is needed to determine whether anesthetic technique influences outcomes in this subgroup.

## 1. Introduction

High-grade glioma (HGG) is the most common primary malignant brain tumor, with an incidence of 3–5 cases per 100,000 people [1,2]. According to the World Health Organization (WHO) 2021 classification, HGGs include several entities, such as IDH-mutant grade III and grade IV astrocytomas, as well as IDH-mutant and 1p/19q-codeleted oligodendrogliomas [2]. A maximal safe surgical resection, along with systemic therapy and radiation, is the mainstay of treatment [3,4]. Despite the application of multimodal therapy regimens and emerging approaches such as targeted therapy, immunotherapy, and tumor-treating fields, the prognosis remains poor [4]. Median survival for grade III gliomas ranges from 24 to 72 months, while for grade IV gliomas, it is only 14 to 16 months [5,6,7].

Perioperative factors, including volative anesthetic agents, may influence oncological outcomes by modulating host immunity [6,7]. Although this has been explored in colorectal and prostate cancers, its relevance in HGG remains unclear and underexplored. Given the poor prognosis of HGG, it is crucial to investigate all potentially modifiable factors, including anesthetic technique. Clinical and preclinical studies suggest that the type of anesthesia may influence patient outcomes with HGGs. Xu et al. found that propofol exposure in glioma cell cultures inhibited proliferation and invasion and induced cell apoptosis [8]. Shi et al. found that sevoflurane promoted glioma stem cell growth [9]. Huang et al. found that patients undergoing glioblastoma (GB) surgery with total intravenous anesthesia (TIVA) had improved OS and fewer postoperative complications compared to those who received inhalational anesthesia (INHA) [3]. On the other hand, other studies have reported conflicting results. A retrospective study showed no significant difference in OS or PFS between patients undergoing surgery under TIVA versus INHA [1]. Similarly, Schmoch et al. found no difference in survival outcomes in patients with IDH-wildtype GBM between the two anesthetic groups [5].

The choice of anesthetic agent during glioma surgery may have implications extending beyond perioperative management, potentially influencing tumor biology and patient outcomes [8,9]. Preclinical studies have demonstrated that propofol exerts antitumor effects by inhibiting glioma cell proliferation, invasion, and migration, primarily through mechanisms such as upregulation of microRNA-218 and suppression of hypoxia-inducible factor 1-alpha (HIF-1α) and matrix metalloproteinase-2 (MMP-2) activity [10,11,12]. Conversely, volatile anesthetics like sevoflurane have been associated with enhanced glioma stem cell expansion and reduced apoptosis, possibly promoting tumor aggressiveness via activation of hypoxia-inducible factors [13]. Additionally, inhalational agents differ from intravenous anesthetics in their modulation of neuroinflammation and immune responses, influencing oxidative stress and cytokine release [14]. These mechanistic differences suggest that anesthetic choice may affect glioma progression and patient survival, underscoring the need for comprehensive evaluation of clinical outcomes associated with different anesthesia techniques.

Despite these findings, most of the available studies are limited by small sample sizes, methodological heterogeneity, and retrospective study designs. To date, no conclusive evidence has established whether anesthetic choice impacts long-term outcomes in HGG patients, which has resulted in a notable gap in the existing literature. To bridge this gap, we performed a systematic review and meta-analysis to compare the impact of total intravenous anesthesia (TIVA) versus inhalational anesthesia (INHA) on overall survival (OS) and progression-free survival (PFS) in patients with high-grade gliomas (HGGs). By integrating evidence from multiple studies, our analysis offers a comprehensive assessment of how anesthetic technique may influence OS and PFS in this patient population. To our knowledge, this is the first meta-analysis specifically addressing this association. The results are intended to support future clinical guidelines and underscore the potential of anesthetic choice as a modifiable factor in optimizing oncologic outcomes for individuals with HGGs.

## 2. Methods

### 2.1. Inclusion and Exclusion Criteria

This systematic review and meta-analysis was conducted in accordance with the Cochrane Handbook for Systematic Reviews of Interventions and adhered to the PRISMA (Preferred Reporting Items for Systematic Reviews and Meta-Analyses) guidelines. Ethical approval from an Institutional Review Board was not necessary, as the analysis was based solely on data extracted from previously published, publicly accessible sources. Eligible studies for inclusion met the following criteria: (1) they were either observational in design (case-control, cohort, or cross-sectional) or randomized controlled trials (RCTs), (2) studies including patients undergoing surgical resection for high-grade gliomas (WHO grade 3–4), (3) studies in which patients received total intravenous anesthesia (TIVA) as the intervention, (4) studies including a comparison group receiving inhalational anesthesia (INHA), and (5) studies reporting at least one of the outcomes of interest: recurrence rate, overall survival, or progression-free survival. Studies were excluded if they met one of the following criteria: (1) studies including patients without high-grade gliomas or not undergoing surgical treatment, (2) studies not reporting total intravenous anesthesia as an intervention, (3) different study designs (case reports, gray literature, case series), (4) studies without an appropriate control group receiving inhalational anesthesia, (5) overlapping population, and (6) studies not reporting any of the predefined outcomes of interest. The protocol for this systematic review and meta-analysis was prospectively registered in the International Prospective Register of Systematic Reviews (PROSPERO) under the registration number “CRD420251047640”.

### 2.2. Database Search and Extraction of Study Data

A comprehensive literature search was conducted across PubMed, Scopus, and the Cochrane Central Register of Controlled Trials from database inception through March 2025, utilizing the following search strategy: (“Glioma” [Mesh] OR “Glioblastoma” [Mesh] OR glioma OR glioblastoma OR “high-grade glioma” OR GBM OR GB OR “anaplastic astrocytoma”) AND (“Anesthesia, Intravenous” [Mesh] OR “total intravenous anesthesia” OR “intravenous anesthesia” OR “intravenous anaesthetics” OR “intravenous anesthetics” OR propofol) AND (“Anesthesia, Inhalation” [Mesh] OR “inhalational anesthesia” OR “inhalative anesthesia” OR “inhaled anaesthetics” OR sevoflurane OR desflurane). The search was limited to articles published in English, and gray literature was excluded. Additionally, reference lists of all included studies were manually screened to identify further relevant publications. Data extraction was independently performed by two authors (P.P. and D.M.) using predefined criteria and quality assessment tools, facilitated by Rayyan software [accessed on 30 May 2025] [15]. Discrepancies between the reviewers were resolved through discussion and consensus.

### 2.3. Outcome Measures and Subgroup Analyses

The meta-analytic approach evaluated OS and PFS as primary endpoints. Additionally, secondary analyses were conducted based on tumor grade, median OS, and PFS values, as well as single-arm meta-analyses using pooled medians for each anesthesia type. We also performed subgroup analyses based on the tumor grade and risk of bias assessment to explore its potential impact on the primary outcomes.

### 2.4. Risk of Bias Assessment

The risk of bias was evaluated using the Cochrane Collaboration’s ROBINS-I tool for non-randomized studies of interventions [16], which classifies bias risk into four levels: low, moderate, serious, and critical. Two reviewers (E.A. and N.E.) independently conducted the assessments, with any disagreements resolved by consensus. Publication bias was examined through funnel plot analysis by plotting study weights against effect estimates. In accordance with Cochrane recommendations, the Egger test was not performed due to the inclusion of fewer than 10 studies in the meta-analysis [17].

### 2.5. Statistical Methods

Hazard ratios (HRs) with corresponding 95% confidence intervals (CIs) were calculated to compare time-to-event outcomes utilizing the restricted maximum-likelihood estimator within a random-effects framework. To address potential demographic and methodological heterogeneity, a random-effects model was employed for all analyses. Between-study heterogeneity was evaluated using the I^2^ statistic alongside the Cochrane Q test. Statistical significance was defined as two-sided *p*-values less than 0.05. To minimize the risk of selection bias, secondary and subgroup analyses were based on tumor grade, risk of bias assessment, median OS and PFS values, as well as single-arm meta-analyses using pooled medians for each anesthesia type. Leave-one-out (LOO) sensitivity analyses were also conducted to assess the robustness of the findings. To derive hazard ratios from KM, we used the validated method described by Liu et al., in which individual patient data was generated using a Shiny application [18]. To calculate the median differences, we applied the Quantile Matching Estimation method described by McGrath et al. [19]. A Baujat plot was created to pinpoint studies that contributed disproportionately to heterogeneity and to assess their impact on the overall meta-analysis results. This graphical tool displays each study’s contribution to heterogeneity on the *x*-axis against its relative weight in the meta-analysis on the *y*-axis, facilitating the identification of outliers or influential studies. Statistical analyses were conducted using R software version 4.3.1, employing the ‘metafor’ and ‘meta’ packages [20].

## 3. Results

### 3.1. Study Inclusion and Baseline Data

**Figure 1:**
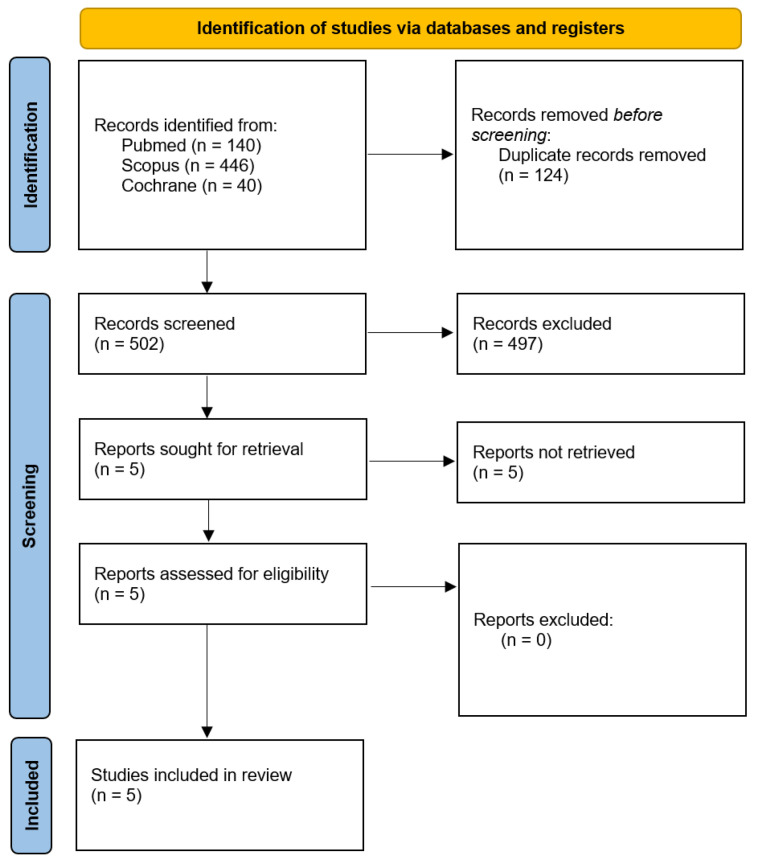
PRISMA flow diagram and study selection.

The literature search identified 626 records. Following the removal of duplicates and exclusion of irrelevant articles and abstracts, five studies remained for full-text review to assess eligibility based on predefined inclusion and exclusion criteria (Figure 1). Ultimately, these five studies, comprising a total of 827 patients, were included [1,2,3,4,5]. The mean age of the study population was 58 years, with females representing approximately 38%. Detailed baseline characteristics of the included populations are summarized in Table 1.

### 3.2. Pooled Analyses of All Included Studies

#### 3.2.1. OS

**Figure 2:**
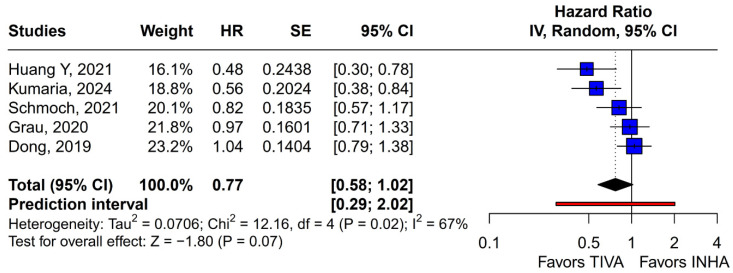
In terms of overall survival, no statistically significant differences were observed [1,2,3,4,5].

**Figure 3:**
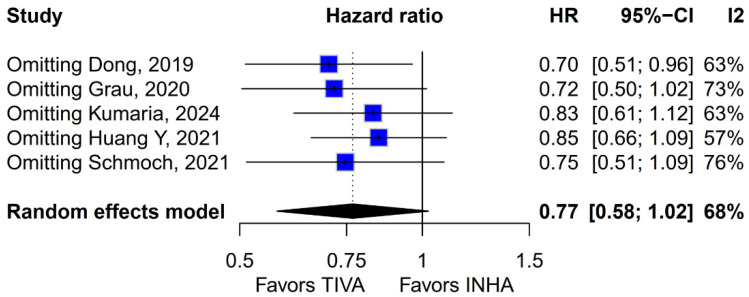
Regarding OS, the overall effect size remained consistent across all iterations and the result remained non-significant in all cases [1,2,3,4,5].

**Figure 4:**
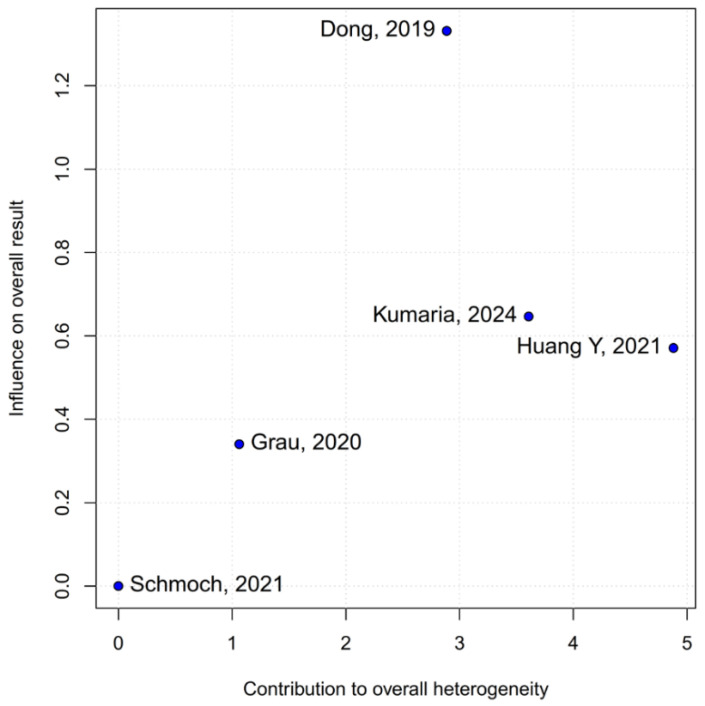
The Baujat plot for OS showing the contribution of individual studies to overall heterogeneity and their influence on the overall meta-analysis results [1,2,3,4,5].

**Figure 5:**
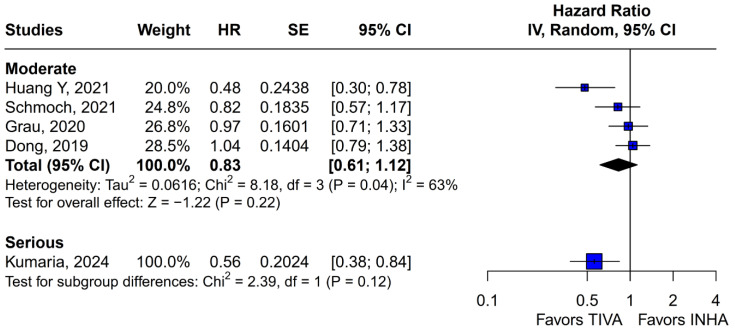
No statistically significant differences emerged based on risk of bias assessment differences between the subgroups [1,2,3,4,5].

No statistically significant differences were observed between the groups (HR 0.77; 95% CI [0.58; 1.02]; *p* = 0.07; I^2^ = 67%) (Figure 2). A LOO analysis was performed to test the robustness of the results. The overall effect size remained consistent across all iterations, and the result remained non-significant in all cases (HR 0.77; 95% CI [0.58; 1.02]; *p* = 0.07; I^2^ = 67%) (Figure 3). This suggests that no single study has a disproportional influence on the overall outcome. The Baujat plot identified the study by Dong (2019) [1] as potentially influential, making a significant contribution to both the overall effect size and heterogeneity (Figure 4). Subgroup analysis stratified by risk of bias revealed no statistically significant differences between the groups (HR 0.83; 95% CI, 0.61 to 1.12; *p* = 0.12; I^2^ = 63%) (Figure 5).

#### 3.2.2. PFS

**Figure 6:**
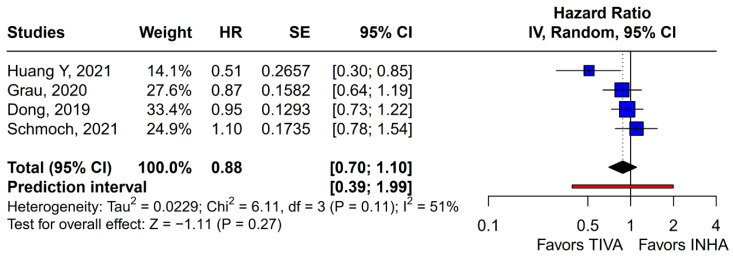
Regarding PFS, no statistically significant differences were observed between the groups [1,2,3,5].

**Figure 7:**
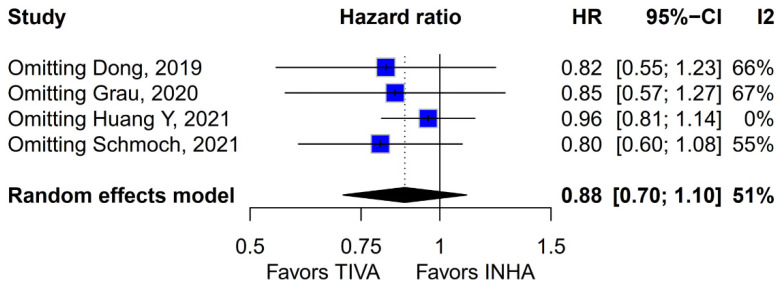
Regarding PFS, the overall effect size remained consistent across all iterations and the result remained non-significant in all cases [1,2,3,5].

**Figure 8:**
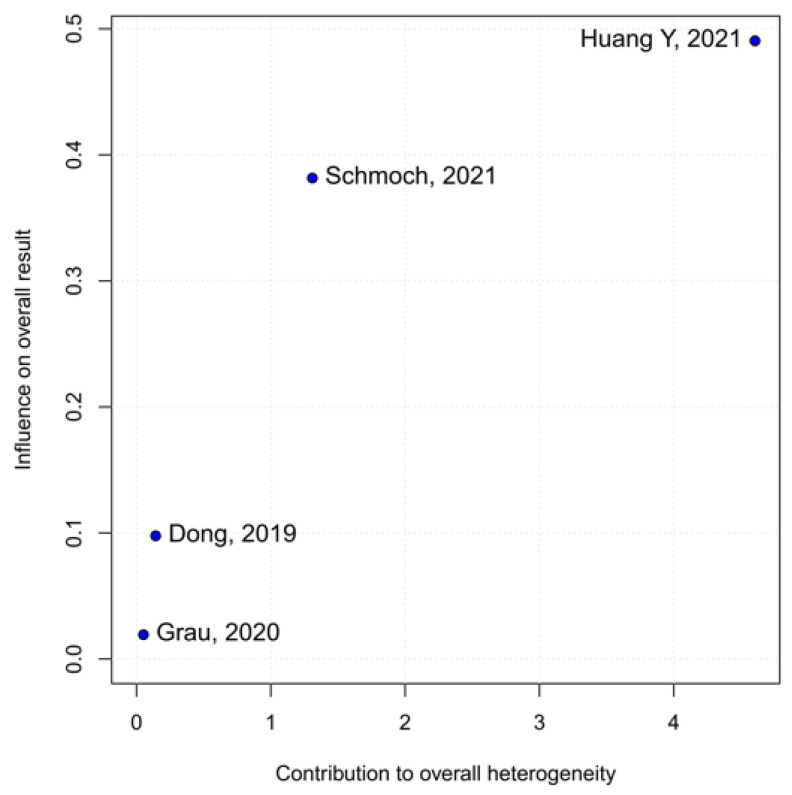
The Baujat plot for PFS showing the contribution of individual studies to overall heterogeneity and their influence on the overall meta-analysis results [1,2,3,5].

No statistically significant differences were observed between the groups (HR 0.88; 95% CI [0.70; 1.10]; *p* = 0.27; I^2^ = 51%) (Figure 6). A LOO analysis was performed to test the robustness of the results. The overall effect size remained consistent across all iterations, and the result remained non-significant in all cases (HR 0.88; 95% CI [0.70; 1.10]; *p* = 0.27; I^2^ = 51%) (Figure 7). This suggests that no single study has a disproportional influence on the overall outcome. The Baujat plot identified the studies by Huang Y (2021) [3] and Schmoch (2021) [5] as potentially influential, contributing substantially to the overall result and heterogeneity (Figure 8).

### 3.3. Secondary and Subgroup Analyses

#### 3.3.1. Median OS (mOS)

**Figure 9:**
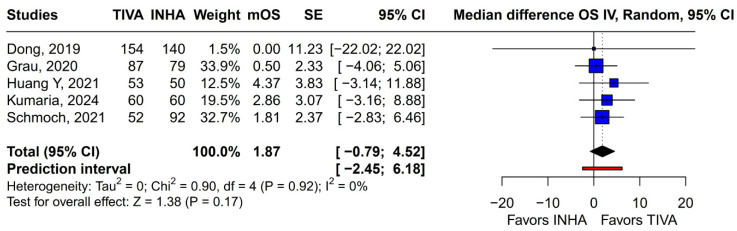
In terms of mOS, there were no statistically significant differences between the groups [1,2,3,4,5].

**Figure 10:**
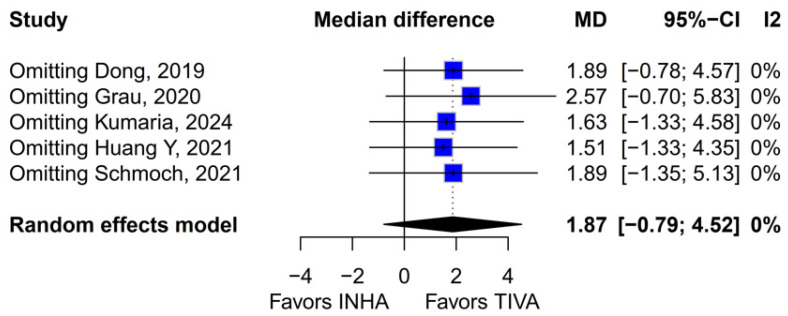
Regarding mOS, the overall effect size remained consistent across all iterations and the result remained non-significant in all cases [1,2,3,4,5].

**Figure 11:**
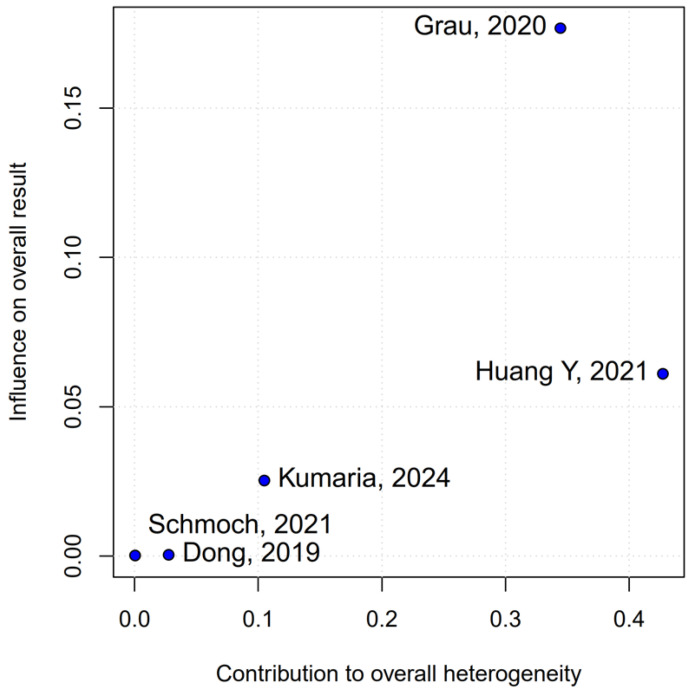
The Baujat plot for mOS showing the contribution of individual studies to overall heterogeneity and their influence on the overall meta-analysis results [1,2,3,4,5].

**Figure 12:**
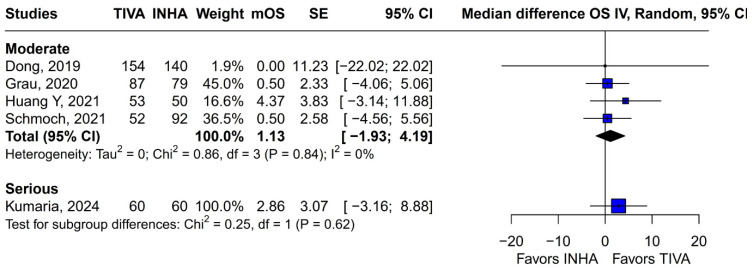
Risk of bias-based subgroup analysis revealed no significant differences between the groups [1,2,3,4,5].

Subgroup analysis based on the median difference (MD) revealed no statistically significant differences between the groups (MD 1.87; 95% CI, −0.79 to 4.52; *p* = 0.17; I^2^ = 0%) (Figure 9). A LOO sensitivity analysis was conducted to assess the stability of the findings. The overall effect size remained consistent across all iterations, with results remaining non-significant throughout (MD 1.87; 95% CI, −0.79 to 4.52; *p* = 0.17; I^2^ = 0%) (Figure 10), indicating that no individual study exerted undue influence on the pooled outcome. The Baujat plot identified the study by Grau et al. (2020) [2] as potentially influential, contributing notably to both the overall effect and heterogeneity (Figure 11). Additionally, subgroup analysis stratified by risk of bias demonstrated no statistically significant differences between the groups (MD 1.13; 95% CI, −1.93 to 4.19; *p* = 0.62; I^2^ = 0%) (Figure 12)).

#### 3.3.2. Single-Arm Meta-Analysis of Pooled mOS

**Figure 13:**
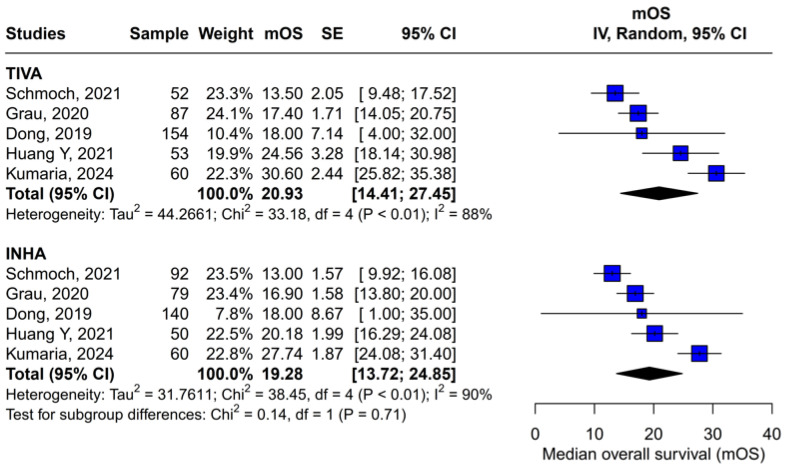
Regarding mOS, there were no statistically significant differences between the subgroups [1,2,3,4,5].

A subgroup single-arm meta-analysis was conducted to evaluate mOS in patients receiving either TIVA or INHA. In the TIVA group, the pooled mOS was 20.93 (95% CI [14.41; 27.45]; I^2^ = 88%). In the INHA group, the pooled mOS was 19.28 (95% CI [13.72; 24.85]; I^2^ = 90%). The test for subgroup differences between TIVA and INHA found no statistically significant differences between the groups (*p* = 0.71) (Figure 13).

#### 3.3.3. Median PFS (mPFS)

**Figure 14:**
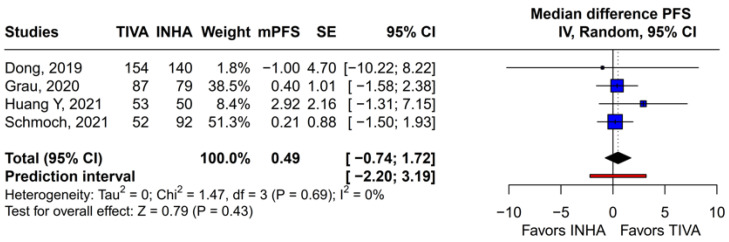
In terms of mPFS, there were no statistically significant differences between the groups [1,2,3,5].

**Figure 15:**
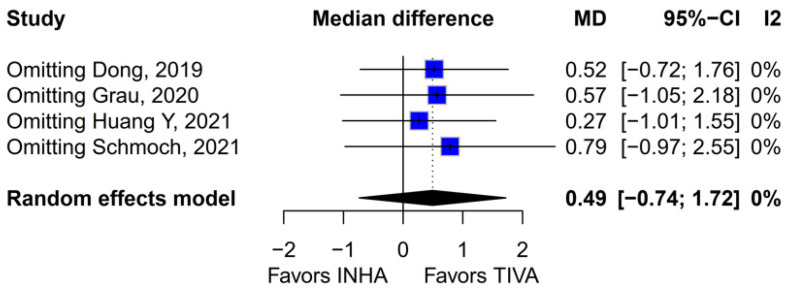
Regarding mPFS, the overall effect size remained consistent across all iterations and the result remained non-significant in all cases [1,2,3,5].

**Figure 16:**
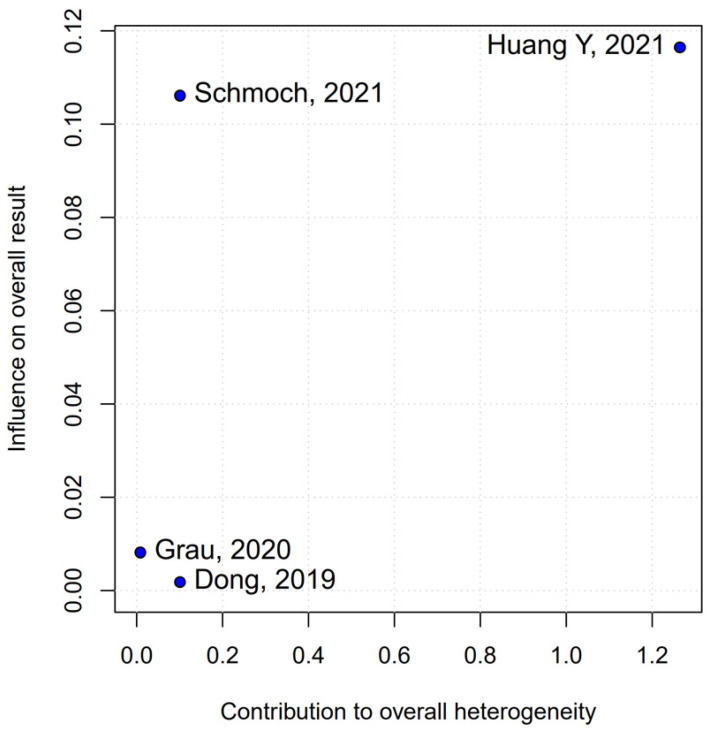
The Baujat plot for mPFS showing the contribution of individual studies to overall heterogeneity and their influence on the overall meta-analysis results [1,2,3,5].

A subgroup analysis based on the median difference (MD) found no statistically significant differences between the groups (MD 0.49; 95% CI [−0.74; 1.72]; *p* = 0.43; I^2^ = 0%) (Figure 14). A LOO analysis was performed to test the robustness of the results. The overall effect size remained consistent across all iterations, and the results remained significant in all cases (MD 0.49; 95% CI [−0.74; 1.72]; *p* = 0.43; I^2^ = 0%) (Figure 15). This suggests that no single study has a disproportional influence on the overall outcome. The Baujat plot identified the studies by Huang Y (2021) [3] and Schmoch (2021) [5] as potentially influential, contributing substantially to the overall result and heterogeneity (Figure 16).

#### 3.3.4. Single-Arm Meta-Analysis of Pooled mPFS

**Figure 17:**
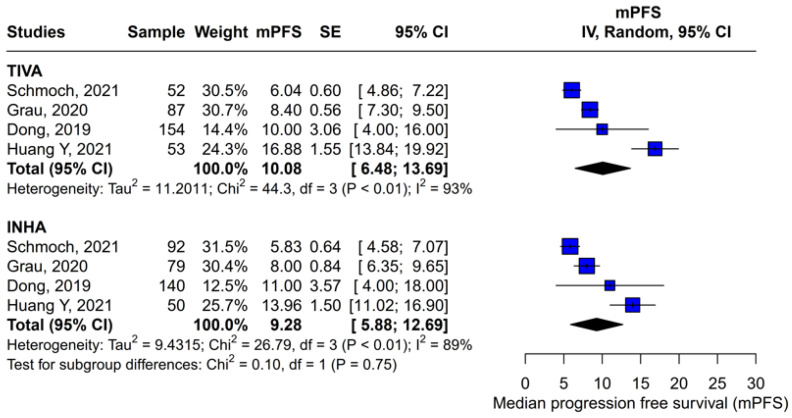
In terms of mPFS, there were no statistically significant differences between the subgroups [1,2,3,5].

A subgroup single-arm meta-analysis was conducted to evaluate the median progression-free survival (mPFS) in patients receiving either TIVA or INHA. In the TIVA group, the pooled mPFS was 10.08 (95% CI [6.48; 13.69]; I^2^ = 93%). In the INHA group, the pooled mPFS was 9.28 (95% CI [5.88; 12.69]; I^2^ = 89%). The test for subgroup differences between TIVA and INHA found no statistically significant differences between the groups (*p* = 0.75) (Figure 17).

#### 3.3.5. Tumor Grade

**Figure 18:**
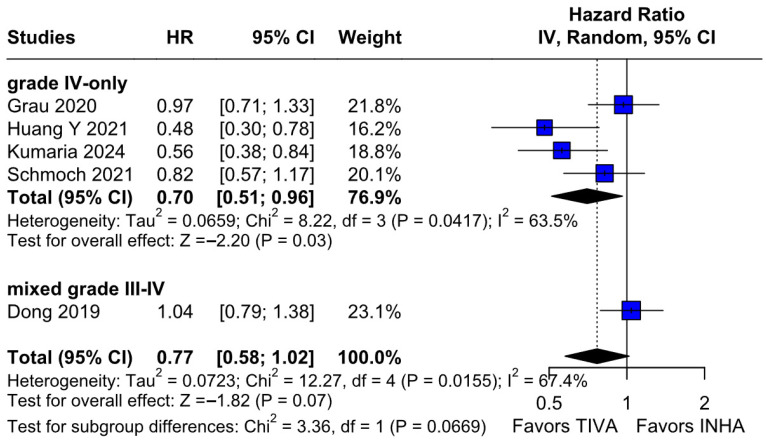
Regarding OS, there was a significant effect in the grade IV-only subgroup, but no significant differences were found between the subgroups [1,2,3,4,5].

**Figure 19:**
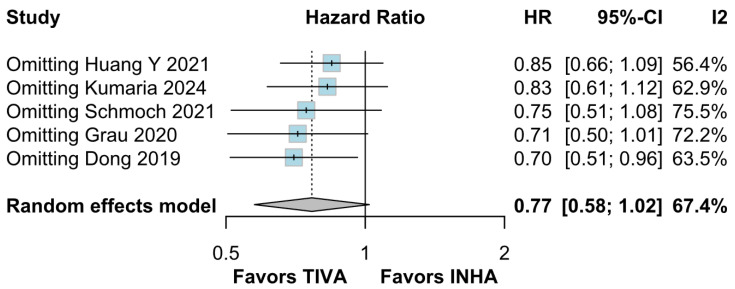
Regarding OS, the overall effect size remained consistent across all iterations and the result remained non-significant in all cases [1,2,3,4,5].

**Figure 20:**
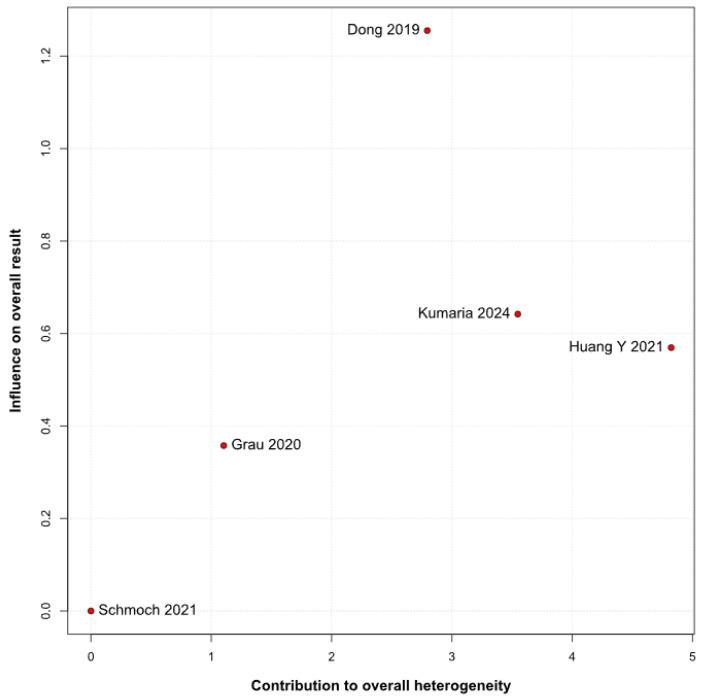
The Baujat plot for OS showing the contribution of individual studies to overall heterogeneity and their influence on the overall meta-analysis results [1,2,3,4,5].

*OS:* In the grade IV-only subgroup, TIVA was associated with a statistically significant improvement in OS compared to INHA (HR 0.70; 95% CI [0.51–0.96]; *p* = 0.03; I^2^ = 63%). In the mixed grade III–IV subgroup, no significant effect was observed (HR 1.04; 95% CI [0.79–1.38]). The test for subgroup differences was not statistically significant (χ^2^ = 3.36, df = 1, *p* = 0.0669), indicating that the observed benefit in grade IV patients may not represent a robust differential effect across tumor grades and should be interpreted with caution (Figure 18). A LOO sensitivity analysis was conducted to evaluate the robustness of the findings. The overall effect size remained stable across all iterations (HR 0.77; 95% CI, 0.58–1.02; I^2^ = 67%) (Figure 19), indicating that no single study exerted a disproportionate influence on the pooled outcome. The Baujat plot identified the studies by Dong (2019) [1], Kumaria (2024) [4], and Huang Y. (2021) [3] as potentially influential contributors to overall heterogeneity (Figure 20).

**Figure 21:**
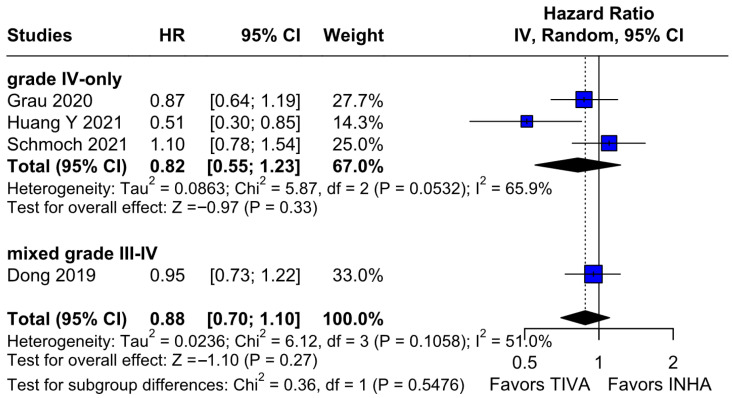
Regarding PFS, no statistically significant differences were observed within each subgroup or between the subgroups [1,2,3,5].

**Figure 22:**
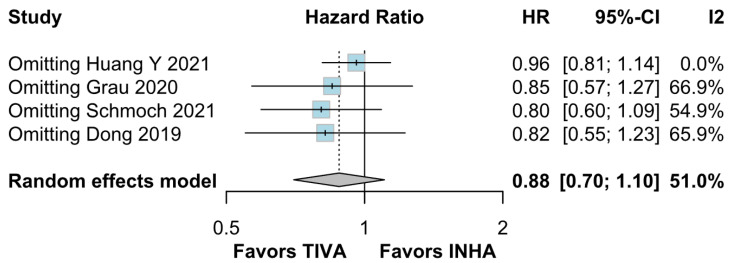
Regarding PFS, the overall effect size remained consistent across all iterations, and the results remained non-significant in all cases [1,2,3,5].

**Figure 23:**
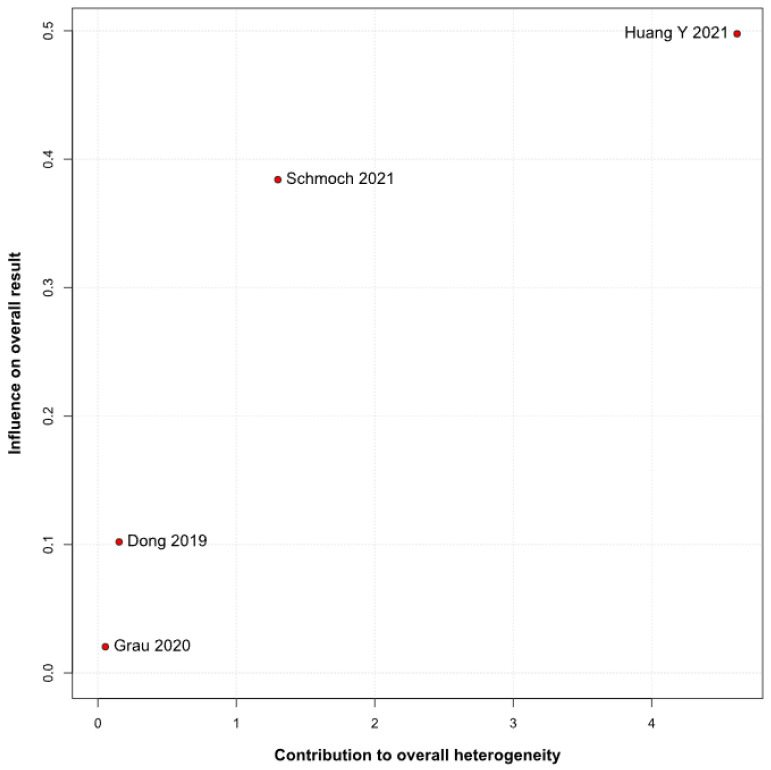
The Baujat plot for PFS showing the impact of individual studies on overall heterogeneity and their influence on meta-analysis outcomes [1,2,3,5].

*PFS*: In the grade IV-only subgroup, there were no statistically significant differences between the groups (HR 0.82; 95% CI [0.55; 1.23]; *p* = 0.33; I^2^ = 65.9%). In the mixed grade III-IV subgroup, there was no significant effect (HR 0.95; 95% CI [0.73; 1.22]. The test for subgroup differences was not statistically significant (χ^2^ = 0.36, df = 1, *p* = 0.5476), indicating no evidence of a differential effect based on tumor grade (Figure 21). A LOO sensitivity analysis was performed to test the robustness of our results. The overall effect size remained consistent across all iterations, and the result remained non-significant in all cases (HR 0.88; 95% CI [0.70; 1.10]; I^2^ = 51%) (Figure 22). These findings indicate that no individual study exerted a disproportionate influence on the overall outcome. However, the Baujat plot highlighted the studies by Schmoch (2021) [5] and Huang Y. (2021) [3] as potentially influential, significantly contributing to both the overall effect and heterogeneity (Figure 23).

### 3.4. Quality Assessment

**Figure 24:**
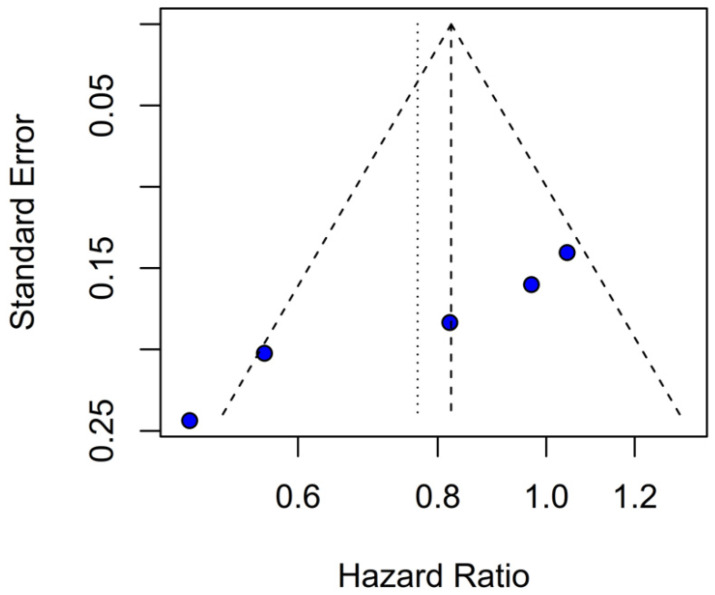
Publication bias evaluation for OS was assessed by plotting individual study weights against point estimates.

**Figure 25:**
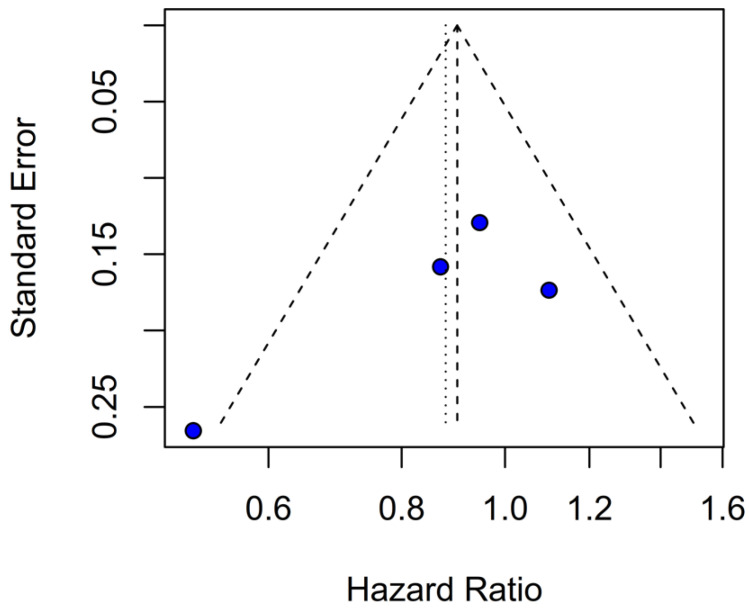
Publication bias for PFS was assessed by plotting individual study weights against their point estimates.

**Figure 26:**
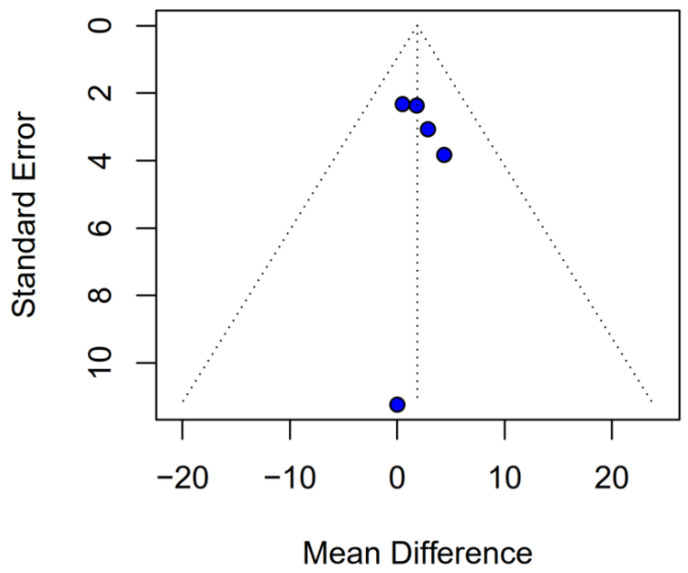
Publication bias for mOS was evaluated by plotting individual study weights against their point estimates.

**Figure 27:**
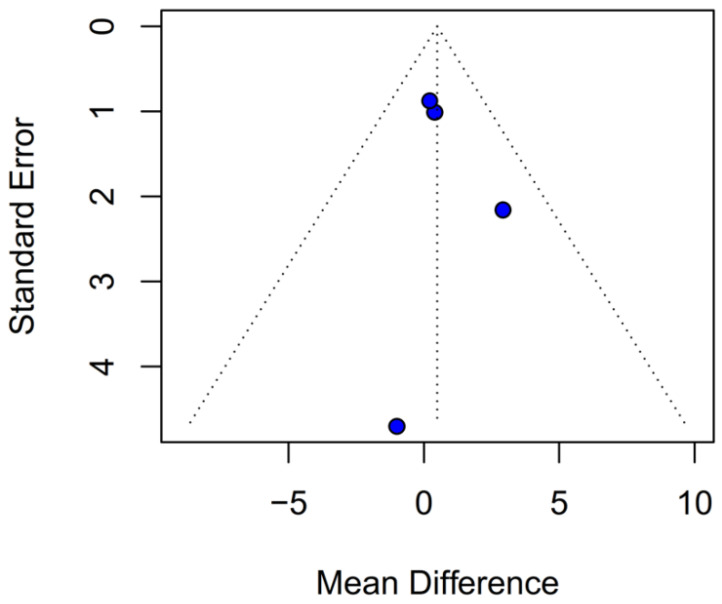
Publication bias for mPFS was assessed by plotting individual study weights against their point estimates.

**Figure 28:**
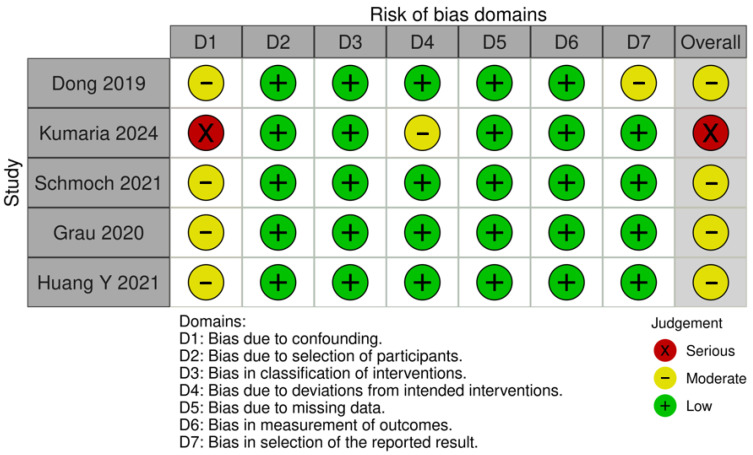
Risk of bias assessment [1,2,3,4,5].

The funnel-plot analyses for HRs of OS and PFS demonstrated a highly asymmetrical distribution of studies, suggesting potential publication bias (Figure 24 and Figure 25). In contrast, funnel plots based on mOS and mPFS indicated only a mild asymmetrical distribution. Therefore, a probable presence of publication bias was identified (Figure 26 and Figure 27) in the five studies included; three were rated as having a moderate risk of bias, while two were classified as having a serious risk of bias according to the ROBINS-I tool. A detailed assessment of each study is presented in Figure 28. The predominant source of bias across studies was confounding, with four studies judged to have a moderate risk and one a serious risk of bias in this domain.

## 4. Discussion

The potential influence of anesthetic technique on long-term oncological outcomes following cancer surgery remains a subject of intense debate and investigation. For patients undergoing resection of gliomas, particularly high-grade gliomas like glioblastoma, where prognosis is often poor despite multimodal therapy, any modifiable factor that could impact survival or recurrence is of significant clinical interest. In this systematic review and meta-analysis of 5 studies and 827 patients, we evaluated the effect of TIVA versus INHA on OS and PFS in HGG patients. The main findings from the pooled analyses were as follows: (1) There were no statistically significant differences between the groups regarding OS or PFS; (2) In terms of mOS or mPFS, there were also no statistically significant differences between the groups; (3) Single-arm meta-analyses of mOS and mPFS for TIVA and INHA also showed no statistically significant subgroup differences. Our review highlights the lack of comprehensive data on the impact of anesthetic technique on long-term outcomes in HGG patients. This meta-analysis aimed to synthesize the available evidence comparing TIVA, typically propofol-based, with INHA regarding OS and PFS in this patient population.

The principal findings of this analysis, encompassing data from 827 patients (406 receiving TIVA, 421 receiving INHA) across five retrospective studies, indicate no statistically significant difference between the two anesthetic techniques for either primary endpoint. Specifically, the pooled HR for OS was 0.77 (95% CI [0.58; 1.02]; *p* = 0.07), and for PFS, the HR was 0.88 (95% CI [0.70; 1.10]; *p* = 0.27). While the point estimate for OS suggested a potential 23% reduction in the hazard of death associated with TIVA, the result did not achieve conventional statistical significance, and the confidence interval included the possibility of no difference. The PFS results more clearly indicated equivalence between the groups. However, these summary estimates must be interpreted with considerable caution due to the substantial heterogeneity observed, particularly for OS (I^2^ = 67%), and moderate heterogeneity for PFS (I^2^ = 51%), alongside indications of publication bias. Notably, subgroup analysis by tumor grade revealed a statistically significant survival benefit associated with TIVA in grade IV tumors (HR 0.70; 95% CI [0.51–0.96]; *p* = 0.03). However, no significant effect was observed in the mixed grade III–IV subgroup. Although the test for subgroup differences did not reach significance (*p* = 0.0669), this trend suggests that tumor grade may influence anesthetic effect and highlights the need for stratified analysis in future studies.

The pooled analysis of OS yielded an HR of 0.77 in favor of propofol-based TIVA versus INHA, with a *p*-value of 0.07, just shy of conventional significance. However, inconsistency across studies was high (I^2^ = 67%), indicating that the true effect likely varies from one setting or patient population to another. Such heterogeneity means that the pooled HR may not accurately reflect a uniform benefit of TIVA for all glioma patients. This is further supported by the grade-specific analysis, which demonstrated a significant benefit of TIVA in grade IV patients, but not in mixed-grade populations—pointing to potential biological or clinical differences between tumor grades that may mediate anesthetic effects. A Baujat plot pinpointed Dong et al. (2019) [1] as a major driver of both the overall effect and heterogeneity. In their cohort of high-grade glioma patients, median OS and PFS did not differ overall between propofol TIVA and sevoflurane INHA (both medians 18 months). Yet, when patients were stratified by preoperative Karnofsky Performance Status (KPS), those with poorer function (KPS < 80) experienced significantly longer OS with propofol (15 vs. 11 months) and a lower risk of death, whereas no difference emerged in the higher-function subgroup [1]. This context-dependent finding—absent from other trials—likely inflated the apparent benefit of TIVA in the pooled estimate. Heterogeneity was further compounded by the study of Huang et al. (2021), which compared propofol TIVA to desflurane-based INHA in glioblastoma surgery and found a marked OS benefit for TIVA (matched HR 0.51; 95% CI 0.29–0.89; *p* = 0.011) both before and after propensity matching [3]. In contrast, Grau et al. (2020) and Schmoch et al. (2021)—each comparing TIVA to sevoflurane INHA in similar glioblastoma populations—reported no significant OS differences, and Kumaria et al. (2024) likewise observed equivalent survival between propofol/remifentanil TIVA and sevoflurane INHA groups [2,4,5]. These contradictory outcomes underscore that the specific inhalational agent matters: desflurane and sevoflurane differ in their biochemical properties and potential tumor-modulating effects, so grouping all volatile agents into one category injects clinical and biological variability into the meta-analysis.

Beyond heterogeneity, the funnel plot for OS was markedly asymmetric, suggesting the presence of publication bias. Specifically, smaller studies showing no difference—or even favoring INHA—may have gone unpublished or remained inaccessible, skewing the available literature toward positive findings for TIVA. This selective publication may inflate the apparent benefit of TIVA in the pooled analysis and contribute to the non-significant trend observed (HR 0.77; *p* = 0.07). Such bias threatens the validity of the conclusions by systematically distorting effect estimates away from the null, especially when fewer studies are available. The clinical implication is that the observed trend favoring TIVA should be interpreted with caution, as the true effect may be closer to null than suggested by the current data. Likewise, subgroup analysis by risk of bias did not substantially alter the overall effect (I^2^ = 63%), indicating that study quality alone does not account for the observed variability. The single-arm meta-analysis estimated median OS at 20.93 months for TIVA and 19.28 months for INHA (*p* = 0.71), but extreme heterogeneity within each arm (I^2^ = 88% for TIVA, I^2^ = 90% for INHA) renders these values illustrative at best. Together, the potential presence of publication bias and underlying clinical heterogeneity underscore the need for well-powered prospective studies with pre-registered protocols that include both positive and null results.

The PFS found an HR of 0.88 (95% CI 0.70–1.10; *p* = 0.27), indicating no statistically significant difference between propofol-based TIVA and volatile INHA. The confidence interval includes 1.0 comfortably, suggesting equivalence in the risk of disease progression or death when data are aggregated. Moderate statistical heterogeneity was observed (I^2^ = 51%), implying that roughly half of the variation in the study results is due to real differences rather than chance. A Baujat plot identified Huang et al. (2021) [3] and Schmoch et al. (2021) [5] as the most influential contributors to this heterogeneity. In particular, Huang et al. reported a significant reduction in postoperative tumor recurrence for TIVA versus desflurane INHA (matched HR 0.60; 95% CI 0.37–0.97; *p* = 0.040) [3]. This contrasts sharply with trials comparing TIVA to sevoflurane. Similarly, Grau et al. (2020) compared propofol TIVA to predominantly sevoflurane INHA and found virtually identical median recurrence-free survival (RFS): 8.4 months in the TIVA group versus 8.0 months in the INHA group [2]. Dong et al. (2019) similarly reported no significant difference in median PFS (10 months for TIVA vs. 11 months for sevoflurane) [1]. Schmoch et al. (2021) also detected no difference in the time to progression between propofol TIVA and sevoflurane INHA [5]. The clear benefit seen with desflurane in Huang et al. thus appears to drive much of the heterogeneity when pooled with sevoflurane comparisons.

Assessment of publication bias via the funnel plot showed mild asymmetry, suggesting that smaller or negative studies may be underrepresented, though the bias is less pronounced than for OS. LOO sensitivity analyses confirmed that the pooled HR for PFS remained stable when any single study was omitted, indicating that no one trial unduly skewed the overall estimate. A subgroup analysis using MD rather than HR likewise showed no significant effect: MD 0.49 months (95% CI –0.74 to 1.72; *p* = 0.43) with zero heterogeneity (I^2^ = 0%). However, this metric examines a different aspect of the data and does not eliminate the variability seen in HR analyses. The single-arm meta-analyses estimated a pooled median PFS of 10.08 months for the TIVA cohorts and 9.28 months for the INHA cohorts (*p* = 0.75). However, within-arm heterogeneity was extremely high (I^2^ = 93% for TIVA, I^2^ = 89% for INHA), making these median values illustrative at best rather than definitive.

This meta-analysis of retrospective studies found no statistically significant difference between propofol-based TIVA and INHA for either OS or PFS in glioma resection. For OS, there was a non-significant trend favoring TIVA (HR 0.77; *p* = 0.07) accompanied by high heterogeneity (I^2^ = 67%) and funnel-plot asymmetry, suggesting publication bias [1,3]. PFS results were more clearly equivalent (HR 0.88; *p* = 0.27) with moderate heterogeneity (I^2^ = 51%), and leave-one-out sensitivity analyses confirmed the robustness of the null finding [2,5].

The divergence of Huang et al. (2021) [3]—who compared TIVA to desflurane and found significant OS and PFS benefits (matched OS HR 0.51; *p* = 0.011; matched PFS HR 0.60; *p* = 0.040)—from other studies using sevoflurane suggests that the choice of volatile agent drives much of the heterogeneity and may account for any apparent TIVA advantage [3]. Dong et al. (2019) further demonstrated that TIVA may benefit patients with poorer preoperative function (KPS < 80) even when compared to sevoflurane (median OS 15 vs. 11 months) [1]. No survival differences were seen in comparisons of TIVA versus sevoflurane by Grau et al. (2020), Schmoch et al. (2021), or Kumaria et al. (2024) [2,4,5]. The high heterogeneity, potential publication bias, and retrospective design limit the strength of conclusions, though operative efficiency benefits with TIVA (shorter operative times: 104 vs. 129 min) may still inform anesthetic choice in practice [4].

Subgroup analyses by tumor grade added further nuance to our findings. While the overall pooled analysis showed no significant difference in OS between TIVA and INHA, patients with grade IV gliomas experienced a statistically significant OS benefit with TIVA. This effect was not observed in mixed grade III–IV cohorts, and the test for subgroup differences did not reach significance (*p* = 0.0669). Thus, this result should be interpreted with caution, as the observed effect in grade IV tumors may reflect within-group variability rather than a true differential effect across tumor grades. Nonetheless, the finding supports the hypothesis that the benefit of TIVA may be confined to higher-grade tumors, possibly due to differences in tumor biology or treatment responsiveness. Future prospective studies should incorporate stratified analyses by tumor grade to validate this effect.

The present meta-analysis combines data from 827 glioma resection patients and employs rigorous methods—heterogeneity assessment, publication-bias evaluation, and LOO sensitivity analyses—to address a focused clinical question. However, its findings are tempered by several key limitations:

All included studies were retrospective in design, which introduces inherent methodological limitations, including selection bias, unmeasured confounding, and lack of standardization across cohorts. One important source of bias is confounding by indication, as the choice of anesthetic technique may have been influenced by patient-specific factors (e.g., comorbidities, functional status), tumor characteristics (e.g., location, grade), or institutional preferences and protocols. These influencing factors are often not fully reported or controlled for in retrospective analyses. Even in studies that used propensity score matching to balance baseline characteristics, residual confounding cannot be ruled out, particularly for variables not included in the matching process. This limitation is fundamental and restricts the ability to draw causal inferences between anesthetic modality and survival outcomes. As such, our findings should be interpreted as exploratory and hypothesis-generating rather than definitive. Future prospective, randomized studies are needed to establish causality and isolate the effect of anesthetic choice from underlying confounders.

An important limitation of this meta-analysis is the aggregation of different volatile anesthetics, which may have distinct pharmacologic and biologic effects. For instance, sevoflurane and desflurane differ in their mechanisms of action, neuroinflammatory profiles, and reported impacts on tumor biology. Pooling these agents under a single INHA group may contribute to the observed statistical heterogeneity and could mask agent-specific effects. In particular, the substantial heterogeneity observed for OS (I^2^ = 67%) raises concerns about the appropriateness of combining these agents statistically. This heterogeneity may compromise the validity of the pooled estimates and limit the generalizability of the findings to clinical decision-making. Future meta-analyses and prospective trials should perform stratified analyses by specific volatile agents to delineate their individual impact on glioma outcomes and guide more tailored anesthetic strategies.

In addition to methodological concerns, there is growing evidence that different anesthetic agents may exert distinct biological effects on glioma cells and the tumor microenvironment. Preclinical studies suggest that propofol may inhibit glioma cell proliferation, invasion, and migration and promote apoptosis through mechanisms such as the upregulation of microRNA-218 and suppression of HIF-1α and MMP-2 activity [8,11]. In contrast, sevoflurane has been shown to enhance glioma stem cell expansion by activating hypoxia-inducible factors and to suppress apoptosis, thereby potentially promoting tumor aggressiveness [9,12]. Additionally, volatile agents such as sevoflurane and desflurane differ in their neuroinflammatory and immunomodulatory profiles, with evidence indicating that they modulate oxidative stress, cytokine release, and immune cell activity in distinct ways [13,14]. These mechanistic differences may underlie the variable clinical outcomes observed in studies comparing total intravenous anesthesia with inhalational anesthesia and highlight the importance of agent-specific investigation in future research. Clarifying these biologic effects could provide critical insight into how anesthetic choice may influence glioma progression and survival.

Incomplete adjustment for prognostic factors—such as MGMT methylation, IDH status, histological subtype, and extent of resection—limits the ability to explore subgroup effects or perform fully adjusted analyses. Moreover, while tumor grade was examined as a subgroup, the lack of consistent reporting and stratification across studies limited our ability to perform more nuanced, grade-specific analyses. Nevertheless, the observed OS benefit in grade IV tumors warrants further exploration in stratified or grade-specific trials. These unaccounted prognostic variables may confound the association between anesthetic technique and survival outcomes, thereby limiting the strength of causal inferences and the validity of conclusions regarding independent anesthetic effects.

Evidence of publication bias, particularly for OS, suggests that the pooled HR may overestimate any true effect, with the real difference likely closer to null. Although the funnel plot for PFS showed only mild asymmetry, the potential for selective reporting cannot be excluded and may still influence effect estimates. This underscores the need for comprehensive reporting of neutral findings in future studies.

These limitations underscore the need for prospective randomized trials stratified by inhalational agent and accounting for key tumor and patient characteristics. We sought to overcome these limitations by applying LOO sensitivity analyses, as well as Baujat and funnel plot analyses, alongside subgroup analyses stratified by risk of bias and tumor grade, and secondary analyses with median OS and PFS values, as well as single-arm meta-analyses using pooled medians for each anesthesia type.

## 5. Conclusions

This meta-analysis of 827 patients found no statistically significant differences in overall or progression-free survival between TIVA and INHA in patients with HGGs. While subgroup analysis showed a statistically significant association between TIVA and OS in patients with grade IV tumors, the test for subgroup differences was not statistically significant. Therefore, this finding should be interpreted with caution and considered hypothesis-generating. Further studies with larger cohorts, stratified by tumor grade, and with longer follow-up are warranted to explore this potential effect and clarify underlying mechanisms.

## Figures and Tables

**Table 1:** Baseline characteristics.

Study	No. Patients	Age *	Female, %	Type of Anesthesia	KPS *	WHO Tumor Grade	Duration of Surgery (h)	Duration of Anesthesia (h)	BMI *
Dong, 2019 [1]	294	TIVA: 52 INHA: 51	TIVA: 37 INHA: 34	TIVA: 154 INHA: 140	80	III–IV	TIVA: 4.1 INHA: 4.7	TIVA: 5 INHA: 5.5	TIVA: 24 ± 3 INHA: 25 ± 4
Grau, 2020 [2]	158	61	50	TIVA: 79 INHA:79	90	IV	TIVA: 3 INHA: 3.4	TIVA: 4.5 INHA: 4.9	N/A
Huang Y, 2021 [3]	103	TIVA: 57 INHA: 58	TIVA: 53 INHA:28	TIVA: 53 INHA: 50	N/A	IV	N/A	N/A	N/A
Kumaria, 2024 [4]	263	60	TIVA: 43 INHA:29	TIVA: 79 INHA: 184	N/A	IV	TIVA: 1.7 INHA: 2.2	TIVA: 2.7 INHA: 4	TIVA: 27.8 INHA: 28.1
Schmoch, 2021 [5]	471	TIVA: 62 INHA: 63	TIVA: 37 INHA: 35	TIVA: 54 INHA: 417	82	IV	N/A	TIVA: 6.6 INHA: 6.1	TIVA: 25.5 INHA: 25.0

* mean age; * mean KPS—Karnofsky Performance Status; * mean BMI—body mass index; TIVA—total intravenous anesthesia; INHA—inhalational anesthesia; N/A—Not reported.

## Data Availability

Not applicable.
